# HIV-1 Transmission Clustering and Phylodynamics Highlight the Important Role of Young Men Who Have Sex with Men

**DOI:** 10.1089/aid.2018.0039

**Published:** 2018-10-12

**Authors:** Ann M. Dennis, Erik Volz, Simon D.W. Frost, A.S. Md. Mukarram Hossain, Art F.Y. Poon, Peter F. Rebeiro, Sten H. Vermund, Timothy R. Sterling, Marcia L. Kalish

**Affiliations:** ^1^ Division of Infectious Diseases, University of North Carolina, Chapel Hill, North Carolina.; ^2^ Department of Infectious Disease Epidemiology and Centre for Outbreak Analysis and Modeling, Imperial College, London, United Kingdom.; ^3^ Department of Veterinary Medicine, University of Cambridge, Cambridge, United Kingdom.; ^4^ Department of Pathology and Laboratory Medicine, Western University, London, Canada.; ^5^ Division of Infectious Diseases, Department of Medicine, Vanderbilt University School of Medicine, Nashville, Tennessee.; ^6^ Department of Epidemiology of Microbial Diseases, Yale University School of Public Health, New Haven, Connecticut.

**Keywords:** molecular epidemiology, HIV-1, transmission, phylogeny, men who have sex with men, Southeastern United States

## Abstract

More persons living with HIV reside in the Southern United States than in any other region, yet little is known about HIV molecular epidemiology in the South. We used cluster and phylodynamic analyses to evaluate HIV transmission patterns in middle Tennessee. We performed cross-sectional analyses of HIV-1 *pol* sequences and clinical data collected from 2001 to 2015 among persons attending the Vanderbilt Comprehensive Care Clinic. Transmission clusters were identified using maximum likelihood phylogenetics and patristic distance differences. Demographic, risk behavior, and clinical factors were assessed evaluating “active” clusters (clusters including sequences sampled 2011–2015) and associations estimated with logistic regression. Transmission risk ratios for men who have sex with men (MSM) were estimated with phylodynamic models. Among 2915 persons (96% subtype-B sequences), 963 (33%) were members of 292 clusters (distance ≤1.5%, size range 2–39). Most clusters (62%, *n* = 690 persons) were active, either being newly identified (*n* = 80) or showing expansion on existing clusters (*n* = 101). Correlates of active clustering among persons with sequences collected during 2011–2015 included MSM risk and ≤30 years of age. Active clusters were significantly more concentrated in MSM and younger persons than historical clusters. Young MSM (YMSM) (≤26.4 years) had high estimated transmission risk [risk ratio = 4.04 (2.85–5.65) relative to older MSM] and were much more likely to transmit to YMSM. In this Tennessee cohort, transmission clusters over time were more concentrated by MSM and younger age, with high transmission risk among and between YMSM, highlighting the importance of interventions among this group. Detecting active clusters could help direct interventions to disrupt ongoing transmission chains.

## Introduction

Abetter understanding of HIV transmission dynamics is needed in areas of the Southeastern United States where HIV incidence has not significantly declined. Southern U.S. states are an epicenter of the national epidemic, accounting for nearly 44% of people living with HIV, but only one third of the U.S. population.^1^ The region also faces significant demographic disparities with disproportionate HIV burden among racial/ethnic minorities, in nonurban areas, and among men who have sex with men (MSM).^1^ In addition, increased rates of opiate injection drug use in the region, especially in rural areas, are of concern for rapid dissemination of HIV such as occurred in Scott County, Indiana in 2014.^2^ In Tennessee, new HIV diagnoses remain largely concentrated among MSM.^3^ However, over 43% of counties in Tennessee are at high vulnerability for rapid HIV or HCV dissemination based on composite indicators (i.e., drug overdoses and low income levels) associated with acute HCV infection.^4,5^ Tennessee is one of three states, including West Virginia and Kentucky, with the largest concentration of such vulnerable counties in the United States.^4^ Thus, continued HIV surveillance to direct prevention activities among these high-risk groups is needed.

Molecular epidemiological approaches can help delineate local HIV transmission dynamics, monitor trends in the epidemic, and may assist in the design of targeted interventions. HIV sequences can be used to reconstruct HIV transmission networks or clusters^6^ and reveal links between subepidemics overlapping in geography, time, and social/sexual interaction. Such links are difficult to detect through traditional surveillance such as partner contact tracing, which is resource intensive. With the timely identification of genetic links between subepidemics, targeted interventions, such as increased resource allocation for prevention services employed toward demographic subgroups (geographic or populations), may more effectively curb HIV incidence. However, the success of such interventions hinges on the ability to detect and intervene on new and growing transmission clusters.

Little is known about HIV molecular epidemiology in Tennessee. In this study, our objective was to characterize HIV transmission patterns in a cohort in middle Tennessee through molecular cluster and phylodynamic analyses. We evaluated the degree of local HIV transmission, diversity, and transmission risk behaviors among persons in HIV care in the region through assessment of cluster growth and associations with membership in active clusters that were newly recognized or expanded based on previous clusters. Furthermore, we applied phylodynamic modeling to estimate trends in transmission among MSM.

## Materials and Methods

### Study population

We conducted a retrospective cohort study of persons living with HIV (PLWH) attending the Vanderbilt Comprehensive Care Clinic (VCCC) in Nashville, Tennessee. The catchment area includes metropolitan Nashville and surrounding rural communities and represents an estimated 80% of HIV-positive persons in middle Tennessee who are in active care. The VCCC maintains a clinical and laboratory database with routine abstraction and validation from electronic medical records. Persons were included in this study if they were ≥18 years of age at clinic entry, had ≥1 HIV-1 *pol* sequence sampled from 2001 to 2015, and had ≥2 HIV primary care visits at VCCC within 12 months. The Institutional Review Board at Vanderbilt University approved this study (IRB No. 161368).

### Demographic and clinical variables

We evaluated demographic variables including sex, age and year of diagnosis, race/ethnicity, and country of origin. The area of residence was estimated from three-digit zip codes and distributed in three categories: metropolitan Nashville (code 372), middle Tennessee (surrounding metropolitan Nashville, codes 370 and 371), and other (all other codes). HIV risk behaviors were categorized as MSM, heterosexual, people with history of injection drug use (PWID), and other/unknown. PWID also reporting MSM or heterosexual risk were categorized as PWID. These risk behaviors are patient reported during clinical care and are abstracted from medical records. Clinical data included CD4+ lymphocyte count and HIV-1 RNA viral loads collected ≤60 days of the earliest available sequence and antiretroviral therapy start dates to identify sequences sampled pre-antiretroviral exposure. Pre-therapy genotypes became routine after 2006.^7^

### HIV-1 sequences

Full-length protease and partial reverse transcriptase HIV-1 *pol* sequences were abstracted from drug resistance genotypes performed by LabCorp (Laboratory Corporation of America, Burlington, NC) or the Vanderbilt University Medical Center (all genotypes sampled after October 2010). Most sequences were generated with the ViroSeq HIV-1 Genotyping system. Sequences were aligned using MUSCLE^8^ and edited manually in Bioedit.^9^ Gapped positions were stripped and the final sequence length was 1497 bases. HIV-1 subtypes and recombinants were identified with the Context-based Modeling for Expeditious Typing (COMET HIV-1) tool.^10^

### Cluster analyses

A maximum-likelihood (ML) phylogenetic tree was constructed in FastTree v.2.1.4^11^ with the general time reversible model of nucleotide substitution using the earliest available sequence from each individual. Statistical support of clades was assessed with local support values (Shimodaira-Hasegawa-like test) in FastTree. Pairwise patristic distances (total path length separating tips in the phylogeny) were calculated using a custom Python script^12^ (https://github.com/ArtPoon/bioinfo/blob/master/graphmaker.py). Putative clusters were defined as pairwise patristic distances of ≤1.5% divergence between ≥2 individuals. Of note, sequences linked in clusters do not necessarily represent direct transmission between persons, as unsampled persons may be involved in transmission chain, nor can directionality be inferred. Clusters within the phylogenetic trees were manually examined to ensure sequences from clusters grouped together with high branch support. Cluster diagrams were visualized using Graphviz v.2.38. The distribution of minimum pairwise patristic distances between individuals by various demographic factors were visualized using SinaPlots.^13^

### Statistical analyses

We defined potentially “active” clusters as clusters that included sequences from the most recent sampling timeframe (2011–2015). These clusters could be either newly identified (include only sequences from 2011 to 2015) or represent new growth on an existing cluster (including sequences from 2001 to 2010). Descriptive statistics and bivariate associations were used to assess differences in cluster membership using the chi-squared test for categorical variables and Kruskal-Wallis test for continuous variables. Independent predictors of membership in active clusters were assessed with logistic regression models. A multivariable model was based on inclusion of all variables with *p* < .2 in bivariable analyses. Data were analyzed in Stata 13.0 (StataCorp, College Station, TX).

### Phylodynamic analyses

More recently developed phylodynamic methods can estimate transmission patterns, while accounting for unsampled individuals and variation in sampling times, as well as adjusting for stage of infection at time of sampling, although such methods are more computationally demanding than clustering.

We estimated transmission among MSM using structured population genetic models fitted to dated phylogenies of HIV-1 subtype B^14^ with approaches that have previously been validated by simulations,^15^ and that have been applied to other HIV sequence data.^16^ These methods have also recently been employed among MSM in the United Kingdom.^17^ The models were designed to allow for changing epidemic size and incidence through time, different transmission rates among young versus older MSM, different transmission rates over the course of HIV infection, and different probabilities of transmission between age groups. The model also accounted for importation of HIV lineages into Tennessee from the global HIV reservoir. A compartmental mathematical model was developed in terms of ordinary differential equations, which describe changing epidemic size and incidence among young and older MSM. We defined young MSM (YMSM) as those with an age in the bottom quintile of age distribution at the time of sequencing (threshold 26.4 years of age). A full specification of the mathematical model and diagram representing model structure is provided in the Supplementary Data and Supplementary Table S1 (Supplementary Data are available online at www.liebertpub.com/aid).

A time-calibrated phylogeny was produced by combining the VCCC subtype B sequences with the 2016 reference web alignment of HIV-1 *pol* sequences in the Los Alamos HIV Sequence Database (LANL) (at least 1,000 base pairs long spanning the VCCC alignment, selecting the closest reference to each VCCC sequence). Exact matches (to reduce the possibility that sequences were from the same person) and redundant sequences were removed, yielding 3,283 total sequences (including 486 references); these references were used to estimate the rate of introduction of HIV from outside the sampling area. Including close matches from LANL allows us to distinguish which parts of the phylogeny correspond to virus evolution taking place within Tennessee. An ML tree was reconstructed assuming an SRD06 model of substitution^18^ using IQTREE v.1.5.3.^19^ Tips were dropped if the sampling year was missing. The *treedater* algorithm^20^ was used to obtain a time-calibrated phylogeny, assuming a strict molecular clock and enforcing temporal constraints on the node ordering. As sampling times were only available as years, *treedater* also estimated the sampling dates within each year. As fitting structured coalescent models to trees with over 2000 taxa was not computationally feasible, the phylogeny was divided into four nonoverlapping clades with times to the most recent common ancestor in the early 1980s for downstream analyses.

The mathematical model was converted to a structured coalescent model and fitted to dated phylogenies and associated metadata (age at the time of sampling and CD4+) using the *phydynR* R package (https://github.com/emvolz-phylodynamics/phydynR). The model was fitted using a Bayesian MCMC algorithm implemented in R. The main parameters estimated were the relative risk of transmission among YMSM, the probability that a YMSM will transmit to another YMSM, and the probability that an older MSM will transmit to a YMSM. In addition, we estimated the relative risk of transmission during early HIV infection defined by CD4+ count >500 cells/μL and the rate of importation of HIV lineages from outside Tennessee. A full specification of parameters and a complete description of the model fitting procedure are provided in the Supplementary Data.

## Results

### Study population

In total, 2,915 persons had at least one sequence available. Most persons were male (77.8%), white (47.3%), or black (43.7%), and reported MSM transmission risk (54.8%); 220 (7.6%) were PWID, reported as their primary risk factor (Table 1). The median year of diagnosis was 2004 [interquartile range (IQR) 1999–2009]. At the time of first sequence, most were sampled before 2011 [median year 2010 (IQR 2006–2013)], and 73.6% persons were older than 30 years of age [median age 39 (IQR 30–46) years of age]. Three persons had a first sequence collected between 15 and 17 years of age, before VCCC enrollment at 18 years of age. Nearly all (96%) sequences were subtype B. Among 118 persons with non-B subtypes, a wide diversity of pure subtypes and recombinants were identified; most were subtype C (54.2%), A1 (14.4%), CRF 02_AG (8.5%), CRF 01_AE (7.6%), subtype G (3.4%), other subtypes (4.2%), CRFs (3.4%), or unassigned (4.2%). Of 1,659 persons with country-of-origin data, those with non-B subtypes (*n* = 75) were more likely to be foreign born (84.0% vs. 6.7%; *p* < .001) than persons with subtype B (*n* = 1,477).

**Table 1.** Characteristics of 2,915 Persons with HIV-1 Sequences Sampled at the Vanderbilt Comprehensive Care Clinic, 2001–2015, Stratified by Inclusion in HIV Transmission Clusters at <1.5% Patristic Distance Cutoff

			*In a cluster (*n* = 963)*	
	*Total*	*No cluster*	*Historical*	*Active*
*Characteristic*	n *(%)*	n *(%)*	n *(%)*	n *(%)*
Total, row%	2,915	1,952 (67.0)	273 (9.4)	690 (23.6)
Year of sequence				
2011–2015	1,027 (35.2)	627 (32.1)	—	400 (58.0)
2006–2010	1,159 (38.8)	743 (38.1)	191 (70.0)	225 (32.6)
2001–2005	729 (25.0)	582 (29.8)	82 (30.0)	65 (9.4)
Age at sequence				
≥30 years	2,144 (73.6)	1,586 (81.2)	188 (68.9)	370 (53.6)
<30 years	771 (26.5)	366 (18.8)	85 (31.1)	320 (46.4)
Sex				
Male	2,240 (77.8)	1,464 (75.0)	194 (71.1)	582 (84.4)
Female	675 (23.2)	488 (25.0)	79 (28.9)	108 (15.7)
Race/ethnicity				
White	1,380 (47.3)	934 (47.9)	124 (45.4)	322 (46.7)
Black	1,275 (43.7)	830 (42.5)	133 (48.7)	312 (45.2)
Latino	150 (5.2)	109 (5.6)	7 (2.6)	34 (4.9)
Other/unknown	110 (3.8)	79 (4.1)	9 (3.3)	22 (3.2)
Transmission risk				
MSM	1,597 (54.8)	1,000 (51.2)	127 (46.5)	470 (68.1)
Heterosexual	953 (32.7)	683 (35.0)	115 (42.1)	155 (22.5)
PWID	220 (7.6)	158 (8.1)	28 (10.3)	34 (4.9)
Other/unknown	145 (5.0)	111 (5.7)	3 (1.1)	31 (4.5)
Year of diagnosis				
≥2005	1,208 (41.5)	629 (32.2)	146 (53.5)	433 (62.8)
<2005	1,318 (45.2)	1,070 (54.8)	122 (44.7)	126 (18.3)
Missing	389 (13.3)	253 (13.0)	5 (1.8)	131 (19.0)
Area of residence				
Nashville metro	1,427 (48.9)	951 (48.7)	147 (53.9)	329 (47.7)
Middle Tennessee	1,191 (40.9)	774 (39.7)	105 (38.5)	312 (45.2)
Other	297 (10.2)	227 (11.6)	21 (7.7)	49 (7.1)
CD4 lymphocytes, cells/μL median (IQR)	312 (149–483)	290 (130–470)	306 (136–469)	367 (225–547)
Log_10_ HIV RNA, copies/mL median (IQR)	4.5 (3.8–5.0)	4.4 (3.7–5.0)	4.6 (4.1–5.0)	4.5 (3.9–5.0)
Subtype				
B	2,797 (96.0)	1,858 (95.2)	263 (96.3)	676 (98.0)
Non-B	118 (4.0)	94 (4.8)	109 (3.7)	14 (2.0)

MSM, men who have sex with men; PWID, persons who inject drugs; IQR, interquartile range.

**FIG. 1.**
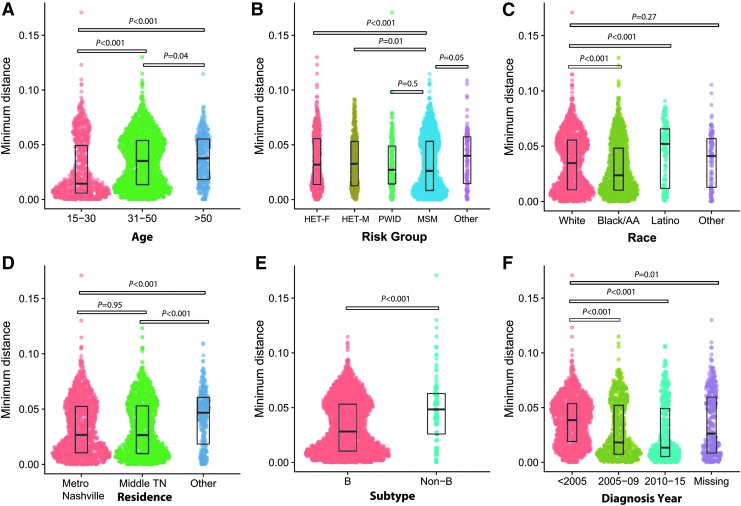
Distribution of the minimum pairwise patristic distances by demographic factor visualized using SinaPlot. *y*-Axis is minimum pairwise distance in nucleotide substitutions per site. Each *dot* represents an individual person. The width of the area corresponds to the density of the data. The median and interquartile range of each distribution are shown with *boxes*. Kruskal-Wallis *p*-values for comparisons are shown. **(A)** Age (years) at the time of first available sequence. **(B)** Transmission risk group categorized as follows: women reporting heterosexual risk only (HET-F), men reporting heterosexual risk only (HET-M), persons who inject drugs (PWID), MSM, and other/unknown risk. *p*-Values shown for comparisons of MSM risk versus each other risk category. **(C)** Race/ethnic group. *p*-values shown for comparisons of whites versus each other risk category. **(D)** Geographic area of residence by 3-digit postal code categorized as follows: metropolitan Nashville (code 372), areas surrounding Nashville metro “Middle Tennessee” (codes 370 and 371), and other (all other codes). **(E)** HIV subtype. **(F)** Year of HIV diagnosis. *p*-Values shown for comparisons of HIV diagnoses before 2005 versus more recent diagnosis. F, female; HET, heterosexual; M, male; MSM, men who have sex with men; PWID, persons who inject drugs. Color images available online at www.liebertpub.com/aid

The distribution of minimum pairwise patristic distances differed between several demographic factors (Fig. 1). Persons <30 years of age at first sequence had lower median minimum distances compared to older persons (0.014 substitutions per site vs. 0.036 for those ≥30 years of age; *p* < .001). Minimum distances were also lower for persons with subtype B compared to those with non-B subtypes, MSM risk compared to women or men reporting heterosexual risk, and black race compared to whites and Latinos, and for those diagnosed during 2005–2015 (all *p* < .001).

### Characteristics of transmission clusters

In the cluster analysis, 33% (*n* = 963) of persons were identified in a cluster based on <1.5% patristic distance (Table 1). There were 292 clusters (size ranged 2–39 persons); 64% were in pairs (*n* = 2), 32% included 3–9 persons, and 4% were ≥10 persons. Most cluster members (*n* = 690, 71.7%) were in 181 active clusters (containing at least one sequence sampled during 2011–2015); the remaining 111 clusters were designated historical clusters (*n* = 273 persons, 28.3%) (The entire network is available in Supplementary Fig. S1). Of 181 active clusters, 101 (55.8%) were growing clusters and 80 (44.2%) were newly recognized clusters (included only sequences sampled during 2011–2015). In bivariable analyses, cluster members differed by persons who were not identified in any cluster by multiple characteristics (Table 1). Members of active clusters were more likely to be younger (46.4% vs. 26.5% were <30 years old) and male (84.4% vs. 75.0%), report MSM risk (68.1% vs. 51.2%), and to be diagnosed more recently (62.8% vs. 41.5% diagnosed ≥2005) compared to persons not in any cluster (all *p* < .001). In addition, active clusters were more likely to be subtype B (98.0% vs. 95.2% not in clusters, *p* = .006). Among persons with first sampling 2011–2015, those with sequences sampled more recently were less likely to be in active clusters (34% of those sampled in 2015 vs. 46% in 2013 were in active clusters). The finding is likely due, in part, to variable sampling density over time; most samples (40%) were collected during 2006–2010 compared to 35% during 2011–2015. For more recent years (i.e., 2014 and 2015 in this dataset), members of clusters may be still be undiagnosed or not yet linked to care, thus less sequences being available.

Cluster composition was assessed by majority risk behavior, race group, youth (defined as majority of cluster members <30 years of age), and area of residence. While most clusters were majority MSM (52.7%; 154/292), active clusters were significantly more likely to be majority MSM (59.7%; *p* < .01). Only four clusters were composed of majority PWID (involving total 30 persons); two were historical (both comprised three men) and two were active clusters (cluster sizes 4 and 20 persons, including >25% women). Among racial composition, most clusters were either majority black (40.8%) or white (39.7%). There was no significant change in composition between active and historical clusters with regard to race; 40.3% active clusters were majority black and 39.8% were majority white (*p* = .9 trend). Active clusters were significantly more concentrated by younger persons. Of active clusters, 36.5% (66/181) were majority <30 years of age compared to all clusters (28.4%; 83/292) and of historical clusters (15.3%: 66/181) (*p* < .001). By area of residence, 24.0% (70/292) of all clusters were entirely composed of residents from metropolitan Nashville, compared to 18.2% of active clusters (*p* < .01). For clusters including persons residing outside of Nashville (*n* = 222), 94% (209/222) included residents of middle Tennessee and 23% (*n* = 51/222) included persons residing outside middle Tennessee. Of the four majority PWID clusters, all included residents from two geographic areas; one cluster included residents from all three regions.

**FIG. 2.**
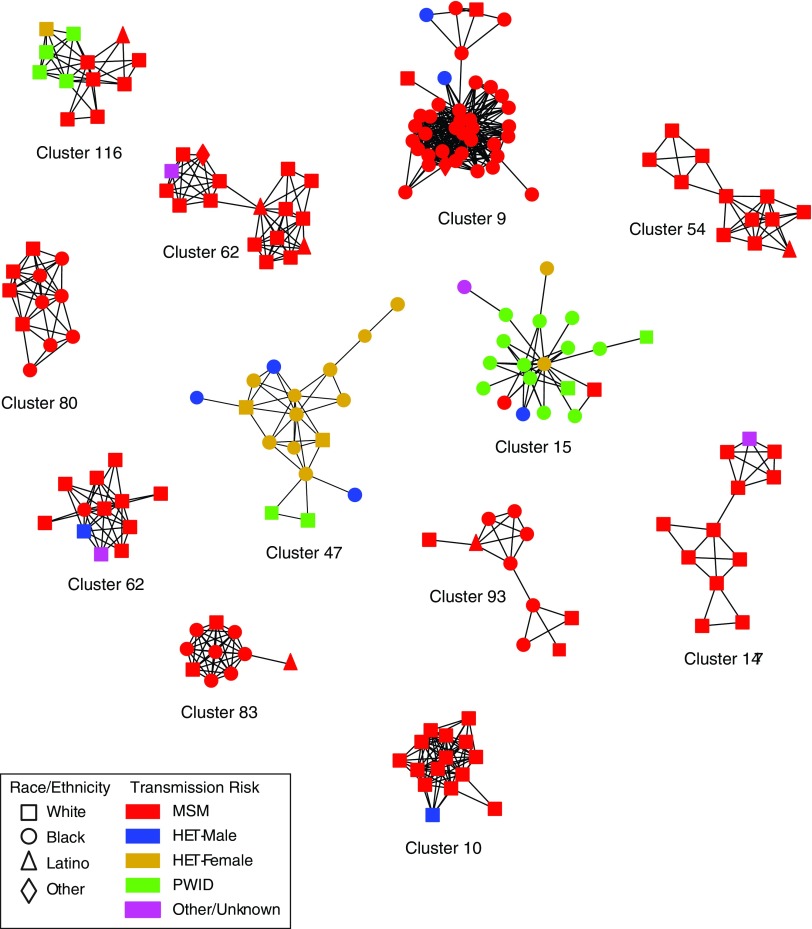
HIV transmission network for clusters involving 10 or more persons inferred from 2,915 partial HIV-1 *pol* sequences sampled at the Vanderbilt Comprehensive Care Clinic, 2001–2015. Nodes indicate individual persons; shapes represent race/ethnicity. Edges indicate linkages ≤1.5% patristic distances. Nodes are color coded by transmission risk group. Color images available online at www.liebertpub.com/aid

Nearly all clusters (95.9%; 280/292) were subtype B, including 12 clusters with ≥10 members (Table 2 and Fig. 2). These large clusters included 187 persons, most were male (87.7%) and 10/12 were majority MSM. Only one large cluster was mostly PWID (Cluster ID 15) and one was majority heterosexual (Cluster ID 47, notably, including 15 women). Of the 12 clusters composed of non-B subtypes, 11 were male-female pairs [subtype C (8), CRF02_AG (2) and CRF01_AE (1)] and one pair were MSM (CRF07_BC).

**Table 2.** Characteristics of 12 Clusters with ≥10 Members Among 2,915 Persons with HIV-1 Sequences Sampled at the Vanderbilt Comprehensive Care Clinic, 2001–2015

*Cluster ID*	*Size*	*M:F*	*Risk (%)*	*Race (%)*	*Age, median (IQR)*	*Residence (%)*	*Sampling period*
Cluster_9	39	39:0	MSM (95)	Black (92)	22 (20–25)	Nashville metro (66)	2006–2015
Cluster_15	20	14:6	PWID (70)	Black (85)	36 (25–44)	Nashville metro (80)	2001–2013
			HET (15)				
			MSM (10)				
Cluster_47	17	3:15	HET (88)	Black (77)	27 (26–30)	Nashville metro (70)	2002–2010
			PWID (12)				
Cluster_67	17	17:0	MSM (94)	White (83)	24 (23–25)	Middle TN (59)	2008–2015
						Nash (29)	
						Other (12)	
Cluster_10	15	15:0	MSM (93)	White (100)	31 (28–42)	Middle TN (60)	2004–2012
Cluster_137	12	12:0	MSM (92)	White (100)	42 (37–45)	Middle TN (58)	2004–2015
Cluster_54	12	12:0	MSM (100)	White (92)	33 (25–34)	Middle TN (50)	2004–2014
						Nashville metro (42)	
Cluster_116	12	9:3	MSM (58), HET (33)	White (92)	32 (23–36)	Middle TN (50)	2004–2015
						Other (50)	
			PWID (8)				
Cluster_62	12	12:0	MSM (83)	White (92)	41 (36–49)	Nashville metro (58)	2002–2013
			PWID (8)				
Cluster_180	11	11:0	MSM (100)	Black (64)	26 (22–39)	Nashville metro (63)	2007–2011
				White (36)			
Cluster_93	10	10:0	MSM (100)	Black (60)	31 (23–41)	Middle TN (70)	2005–2012
				White (30)			
Cluster_83	10	10:0	MSM (100)	Black (70)	20 (19–31)	Nashville metro (80)	2009–2015
				White (20)			

F, female; HET, heterosexual; M, male.

### Correlates of clustering

Among the subset of 1,027 persons with sequence sampled during 2011–2015, we evaluated correlates to membership in active clusters. In total, 400 persons (39%) newly sampled in this later timeframe were in 181 active clusters. In the multivariable analysis, MSM risk, age <30 years of age [odds ratio (OR) = 2.48; 95% confidence interval (CI) 1.85–3.31], higher CD4+ cell counts, and higher log_10_ HIV viral loads were associated with cluster membership (Table 3). Persons of black race, residing outside middle Tennessee, or with sequences sampled in calendar years that are more recent were less likely to be in these active clusters.

**Table 3.** Characteristics Included in the Multivariable Logistic Regression Model of Being in a Cluster, Among 1,027 Persons with Sequences Sampled at Vanderbilt Comprehensive Care Clinic, 2011–2015

*Characteristic*	*Total,* n *(%)*	*In cluster,* n *(%)*	*Multivariable, OR (95% CI)*^a^
Total, row%	1,027	400 (38.9)	
Sex and risk, *n* = *966*			
Female–WSM	179 (17)	50 (13)	Ref
PWID	42 (4)	12 (3)	1.15 (0.52–2.55)
Male–HET	126 (12)	44 (11)	1.57 (0.92–2.66)
Male–MSM	619 (60)	270 (68)	**1.79 (1.21–2.65)**
Race/ethnicity			
White	471 (46)	195 (49)	Ref
Black/AA	473 (46)	176 (44)	**0.71 (0.53–0.97)**
Latino	53 (5)	19 (5)	0.82 (0.44–1.54)
Other/unknown	30 (3)	10 (3)	0.64 (0.28–1.50)
Age			
≥30 years	663 (65)	210 (53)	Ref
<30 years	364 (35)	190 (48)	**2.48 (1.85–3.31)**
Sequence year, median (IQR)	2013 (2011–2014)	2013 (2011–2014)	**0.89 (0.80–0.98)**
Area of residence			
Nashville metro	469 (46)	184 (46)	Ref
Middle Tennessee	463 (45)	190 (48)	1.05 (0.79–1.40)
Other	95 (9)	26 (7)	**0.49 (0.29–0.84)**
CD4 lymphocyte >500, cells/μL	301 (30)	131 (33)	**1.40 (1.03–1.90)**
HIV RNA viral load, median log10 copies/mL (IQR)	4.5 (3.7–5.0)	4.5 (3.9–5.1)	**1.19 (1.03–1.38)**

^a^ All listed variables were retained in the multivariable model: sex/transmission risk, race/ethnicity, age, sequence year, area of residence, CD4 lymphocyte count, and HIV RNA viral load. *Bold* estimates are statistically significant (*p* < .05).

AA, African-American; CI, confidence interval; OR, odds ratio; WSM, women who have sex with men.

### Phylodynamic analyses

**FIG. 3.**
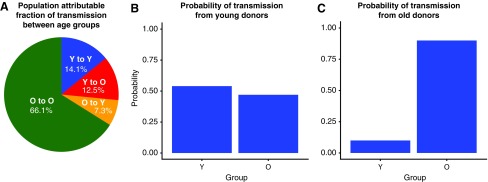
Phylodynamic modeling of 2,792 HIV-1 subtype B partial pol sequences sampled at the Vanderbilt Comprehensive Care Clinic, 2001–2015, to estimate transmission risk ratio for young MSM (Y, age ≤26.4 years) compared to older men (O, age >26.4 years). **(A)** Population attributable fraction of transmission between age groups. **(B)** Probability of transmission from young donors (Y). Each bar represents the proportion of transmissions, which flow from young to each age group. **(C)** Probability of transmission from old donors (O). Each bar represents the proportion of transmissions, which flow from old to each age group. Color images available online at www.liebertpub.com/aid

Phylodynamic modeling of HIV-1 subtype B phylogenies confirmed the importance of YMSM, who are inferred to transmit infection at a higher rate and are more likely to transmit to one another than expected by chance. We estimated the transmission risk ratio for YMSM (≤26.4 years) relative to older MSM to be 4.04 (95% credible interval: 2.85–5.62). YMSM were much more likely to transmit to one another than other MSM (Fig. 3). The probability that a transmission from a YMSM went to another YMSM was 53% (95% CI 39%–70%), despite YMSM comprising only 20% of the sampled population. The probability that a transmission originating in older MSM went to YMSM was 10% (95% CI 7%–15%). We inferred a small net-flow of transmissions from young to old, which lacked statistical significance, in contrast to recent clustering results showing a net flow of transmissions from older to younger MSM.^21^ The ratio of transmissions from young to old versus old to young was 1.7 (95% CI 0.89–2.57). Comparatively, the clustering analysis also showed possible association of YMSM to YMSM transmission. Of 323 YMSM, 201 (62%) were identified in any cluster. Of these, 72% (145/201) were in a cluster with another YMSM (*p* < .001). The phylodynamic model also included a high CD4 compartment (CD4 > 500) to assess risk during early infection. We found only a modest increased risk, which lacked significance [1.27 (95% CI 0.86–1.76)].

We also estimated the rate that lineages flow into Tennessee due to migration of PLWH as well as transmission to Tennessee residents from outside of the state. This rate was estimated to be 0.197 per lineage per year, which implies that the average age of an HIV clade (the direct ancestor of a virus lineage) circulating in Tennessee is ∼5 years. Although it was not the primary aim of phylodynamic modeling, the fitted model provides an estimate of the number of new HIV-1 subtype B infections through time. We estimate that in 2015, there were 475 (95% CI 261–678) new infections, of the same order as the number of reported new HIV cases among MSM (*n* = 433 per year from 2010 to 2015) combined with those with unreported exposure (*n* = 101 in 2015).^22^ Because our coalescent method accounts for incomplete sampling, our results imply that the sampling fraction (proportion of PLWH with a sequence included in the analysis) among MSM was large because the estimated number of new infections is similar to recent estimated number of diagnoses among MSM.

## Discussion

We investigated HIV transmission patterns among persons who received care in middle Tennessee through genetic cluster and phylodynamic analyses with sampling extending 2001–2015, identifying over one third of the study population linked in closely related clusters. Over 60% of clusters were potentially active, including sequences collected between 2011 and 2015, representing new cluster detection or expansion on existing clusters. Younger age and MSM risk were associated with membership in these active clusters, which were also increasingly concentrated among young persons. Integration of phylodynamic modeling confirmed the importance of YMSM, as we estimate a high probability of transmission between YMSM by transmission rate ratios. While we found few clusters concentrated by PWID, many involved MSM and residents outside Nashville, indicating potential bridging between risk groups and regions. Continued surveillance among these high-risk groups with prospective cluster analysis would allow timely detection of new or expanding clusters that could be targeted for intensified prevention.

Genetic cluster and phylodynamic analyses provide information on HIV transmission that would be difficult to ascertain from traditional epidemiological survey data, especially in populations where infection is endemic. Clusters, when integrated with demographic data, provide insight into groups contributing disproportionately to ongoing, localized transmission. Phylodynamics enable more refined estimation of transmission risk, including between groups and locations. Our study is the first characterization of HIV transmission clusters in Tennessee and one of the largest among U.S. studies relying on data from a single clinical cohort. While Southeastern states are at the epicenter of the U.S. HIV epidemic,^1^ few areas in the region investigated local HIV transmission dynamics using molecular epidemiology.^23–25^ Studies based on national molecular HIV surveillance^26–28^ also lack significant representation from southeastern Appalachian states, including Tennessee. These states are areas of concern for increased HIV risk due to concentration of the opioid epidemic.^4^ In Tennessee, HIV transmission remains largely concentrated among MSm^3^ as shown in our study.

We found that younger age is associated with cluster membership and that clusters are becoming increasingly concentrated by younger persons. While this association can be driven by that fact that persons are younger at diagnosis and more likely to be recently infected, our findings correspond with the phylodynamic analysis, which adjusts for stage of infection at the time of sampling. While our analyses unsurprisingly show more clustering among YMSM (following trends in surveillance data), our phylodynamic methods infer patterns of sources and recipients of infection at a population level. In contrast to other reports, which implicated transmission links among disparate age groups suggesting partnerships with older men,^21,28,29^ we estimate a high transmission risk between YMSM. Such findings have implications for prevention by uncovering core groups where interventions could potentially have greater impact.^17,30^ Mixing of different age groups in transmission linkages varies by race/ethnicity; young black and Latino MSM were less likely to have older transmission partners in national molecular surveillance, suggesting these groups are acquiring HIV from other YMSM.^28^ Other U.S. studies also found younger age as a determinant of clustering, including in North Carolina,^23^ Chicago,^31,32^ and Seattle,^21^ and in national molecular surveillance.^26^ Our results harmonize with recent estimates reporting an increase in incidence among MSM aged 25–34 years of age from 2008 to 2014.^33^ A greater proportion of younger persons are estimated to be unaware of their HIV infection (51%, 13–24 years of age, vs. 13%, among PLWH).^34^ In addition, adolescent MSM report more high-risk behaviors compared to heterosexual youth.^35^

We found evidence suggesting assortative mixing by race among transmission clusters. Most clusters were composed of mostly black (40.8%) or white (39.7%) persons, the most prevalent race groups in the study. These findings are congruent with several transmission network studies, including in Mississippi^25^ and North Carolina.^23^ Highly assortative mixing by race/ethnicity was found in U.S. surveillance data, particularly among blacks^26^ and black MSM.^28^ Altogether, these finding suggest that most transmissions occur within race groups, and support tailored interventions addressing risks faced by minority groups.

Further investigation into the geographical extent of HIV transmission networks is important, particularly with regions experiencing increased numbers of PWID. While new HIV diagnoses in Tennessee are largely reported in urban counties and among MSM,^3^ many of these areas are in close proximity to rural counties predicted to be at high risk for an HIV and/or HCV outbreak.^4^ Given that HIV outbreaks among PWID can rapidly occur,^2^ understanding existing network structure between regions can be useful to target prevention resources. Our study is limited by lack of granular geographic data as we analyzed three-digit zip codes, which cover wider-than-optimal geographical areas. Despite this, we found that many clusters contained a person residing outside middle Tennessee, which provides some insight into geographic bridging between regions. Similarly, in Chicago, nearly 72% of clusters of MSM involved several regions.^32^ Among clusters of black YMSM in Mississippi, 52% included residents involving multiple regions.^25^ Further research incorporating more refined geographical and partner contact data for PWID can help better define geographic correlates to transmission.

In addition to clustering-based analyses, which can be readily applied to large sequence datasets, we also conducted a more computationally intensive phylodynamic analysis using structured coalescent models. A major limitation of genetic clustering analyses is that the connections comprising clusters do not have an unambiguous interpretation—for instance, they do not represent transmission events. Phylodynamic models have the advantage of being more robust to sampling bias, as well as providing more mechanistic interpretations, in terms of within- and between-group transmission rates. The results of the phylodynamic analyses were largely concordant with the clustering-based analyses, reinforcing the role of YMSM in the epidemic in middle Tennessee. Phylodynamic analyses also demonstrated that valuable results on transmission risk can be extrapolated from sequence data, despite frequent imports of HIV into this region. Transmission rates between major metropolitan areas could be estimated using similar methodology. In this study, we selected background samples from LANL to provide a diverse background to identify linkages that were likely imported into Tennessee as opposed to circulating endogenously.

Our findings should be interpreted in light of the inherent limitations of HIV phylogenetic analyses. In this study, samplings bias was possible because sequences were only from persons engaged in clinical care, who had a genotype available, which gives an incomplete view of transmission networks. In addition, directionality and direct linkage of transmission cannot be ascertained because additional unsampled parties may be involved in the transmission chain; however, phylodynamic analysis did not require inference of directionality or directness. While information on timing of infection can be used to assist inference of directionality, only weekly informative data in the form of CD4+ counts were available. Furthermore, we did not have infection or seroconversion dates and clusters were defined by sampling dates, which limits confirmation of our conclusions. Most individuals are identified relatively late in infection, and comparison of the VCCC sequences with publicly available data using a phylodynamic model highlighted the high rate of introduction of infections from outside the sampling area.

Combined with epidemiologic surveillance data, phylodynamic and transmission network analyses have the potential to inform HIV prevention. While HIV sequence data are increasingly available and being used to identify HIV genetic clusters, most of these studies are done retrospectively^6^ and lack phylodynamic models. Prospective analyses are needed to test whether identification of putative subepidemics can help guide surveillance and prevention efforts.^36^

## Supplementary Material

Supplemental data

Supplemental data

## Sequence Data

HIV-1 *pol* sequences used in this study are available in GenBank (accession nos.: MH352627–MH355541).
