# Supplementary material for: HIV-1 Transmission Clustering and Phylodynamics Highlight the Important Role of Young Men Who Have Sex with Men
Source: AIDS Res Hum Retroviruses. 2018 Oct 12;34(10):879–88. doi: 10.1089/aid.2018.0039 (PMC6204570; doi:10.1089/aid.2018.0039)
Supplement: Supplemental data [file Supp_Data.pdf]

Supplementary Data

Supporting Methods

Mathematical model

All codes used to specify the mathematical model and estimate model parameters in R can be found at https://github.com/emvolz-phylodynamics/phydynR-tennessee.

The following differential equations specify the evolution of the number of young MSM (YMSM) through time according to a structured SIR model:

$$\dot{Y}_0 = \beta(p_{YY}Y + p_{YI}I)S/N - \gamma_0 Y_0 - \mu_y Y_0$$
$$\dot{Y}_1 = \gamma_0 Y_0 - \gamma_1 Y_1 - \mu_y Y_1$$
$$\dot{Y}_2 = \gamma_1 Y_1 - \gamma_2 Y_2 - \mu_y Y_2$$

Where $Y = Y_0 + Y_1 + Y_2$ is the total number of infected YMSM, Y_0 is the number of early HIV-infected YMSM, Y_1 is the number of chronic YMSM, and Y_2 is the number of AIDS YMSM.

Similarly, $I = I_0 + I_1 + I_2$ is the total number of older MSM infected and I_i is the number of older MSM infected in stage of infection i.

The variables Υ_i are the rates that a host progresses from stage of infection i. These rates were not estimated and were fixed to values based on Cori *et al.* (2015).[S1]

μ_y describes the rate of aging from the younger to the older age groups.

The variables p_{ij} are the proportion of transmissions originating from age group i, which go to age group j.

The variables S and N correspond to the number susceptible and total population size, but note that these are only nuisance parameters for calibrating how incidence changes through time and that this model lacks sufficient realism to estimate the real number susceptible.

The dynamics for infected older MSM are

$$\dot{I}_0 = \beta(p_{YI}Y + p_{II}I)S/N - \gamma_0 I_0 + (\mu_y Y_0 - \mu_I I_0)$$
$$\dot{I}_1 = \gamma_0 I_0 - \gamma_1 I_1 + (\mu_y Y_1 - \mu_I I_1)$$
$$\dot{I}_2 = \gamma_1 I_1 - \gamma_2 I_2 + (\mu_y Y_2 - \mu_I I_2)$$

Where μ_I describes the rate of natural mortality in the older age group.

This model was elaborated in several ways to estimate specific transmission risk for different age groups and stages of infection. The proportion of infections originating from a host in stage of infection i and age group x is proportional to

$$w_{ix} = \omega_i \psi_x$$

Where ω and ψ parameters estimated in the phylodynamic analysis.

In addition, to account for importation of lineages between Tennessee and the global HIV reservoir, we include a symmetric migration process such that young infected hosts in stage i migrate to and from Tennessee at the rate ιY_i and ι is an estimated parameter. A similar process describes migration in the older age group. The symmetric migration process does not influence the epidemic trajectory, but is important for modeling HIV phylogenies with the structured coalescent models since much of the evolutionary history occurs outside of Tennessee.

Model Fitting Procedure

Dated HIV-1 subtype B phylogenies contained more than 2,000 lineages, including global reference sequences, and computation of likelihoods with structured coalescent models was not computationally feasible with the whole dataset. To reduce computation time, we divided the phylogeny into four nonoverlapping clades with time of the most recent ancestors in the early 1980s. Likelihoods were computed in parallel for each clade and log likelihoods summed to produce a likelihood for the entire data set. Three distinct SIR parameters were estimated for each of four clades (initial number infected, population size, and transmission rate), giving a total of 12 parameters specifying the SIR trajectories in all clades. Other parameters that describe lineage importation, transmission risk, and transmission patterns were shared between clades.

The model was fitted by Bayesian MCMC making use of 10 Markov chains running parallel for 20,000 iterations and discarding the first 8,000 iterations from each chain as burn-in. Parameters and prior distributions are provided in Supplementary Table S1. The MCMC utilized a Metropolis Hastings algorithm with symmetric univariate normal proposal distributions. Convergence was assessed by computing effective sample sizes, which exceeded $n = 213$ for all parameters.

Diagram representing structure of mathematical model fitted to dated HIV phylogenies. *Solid arrows* represent progression of natural history of HIV infection. *Dashed lines* represent transmission between different categories of infected hosts. The model includes two overall categories of hosts: YMSM and older MSM. YMSM in all stages of infection also age into the older category (not shown).

Reference

S1. Cori A, Pickles M, van Sighem A, *et al.*: CD4+ cell dynamics in untreated HIV-1 infection: overall rates, and effects of age, viral load, sex and calendar time. AIDS 2015;29:2435–2446.

Supplementary Table S1. Estimated Parameters and Prior Distributions for Mathematical Model Fitted to Dated HIV-1 Subtype B Phylogenies and Associated Metadata (Age and CD4 at the Time of Sampling)

Parameter	*Prior distribution*	*Units*
Relative transmission risk YMSM	Lognormal(log mean = log(1), log sd = 1)	Dimensionless
Relative transmission risk chronic: EHI	Lognormal(log mean = log(1), log sd = 2)	Dimensionless
Lineage importation rate	Exponential (rate = 1/20)	Imports per year per lineage
Probability of transmission from YMSM to other MSM	Beta(2,2)	Dimensionless
Probability of transmission from older MSM to older MSM	Beta(2,2)	Dimensionless
Initial number infected at start of simulations	Exponential(rate = 1)	Number of hosts
Initial susceptible population size	Lognormal(log mean = log(1,000), log sd = 2)	Number of hosts
Transmission rate	Lognormal(log mean = log(1/10), log sd = 1)	Transmissions per infected host per year

EHI, early HIV infected; MSM, men who have sex with men; YMSM, young MSM.
