# Supplementary figures and images for: HIV-1 Transmission Clustering and Phylodynamics Highlight the Important Role of Young Men Who Have Sex with Men
Source: AIDS Res Hum Retroviruses. 2018 Oct 12;34(10):879–88. doi: 10.1089/aid.2018.0039 (PMC6204570; doi:10.1089/aid.2018.0039)

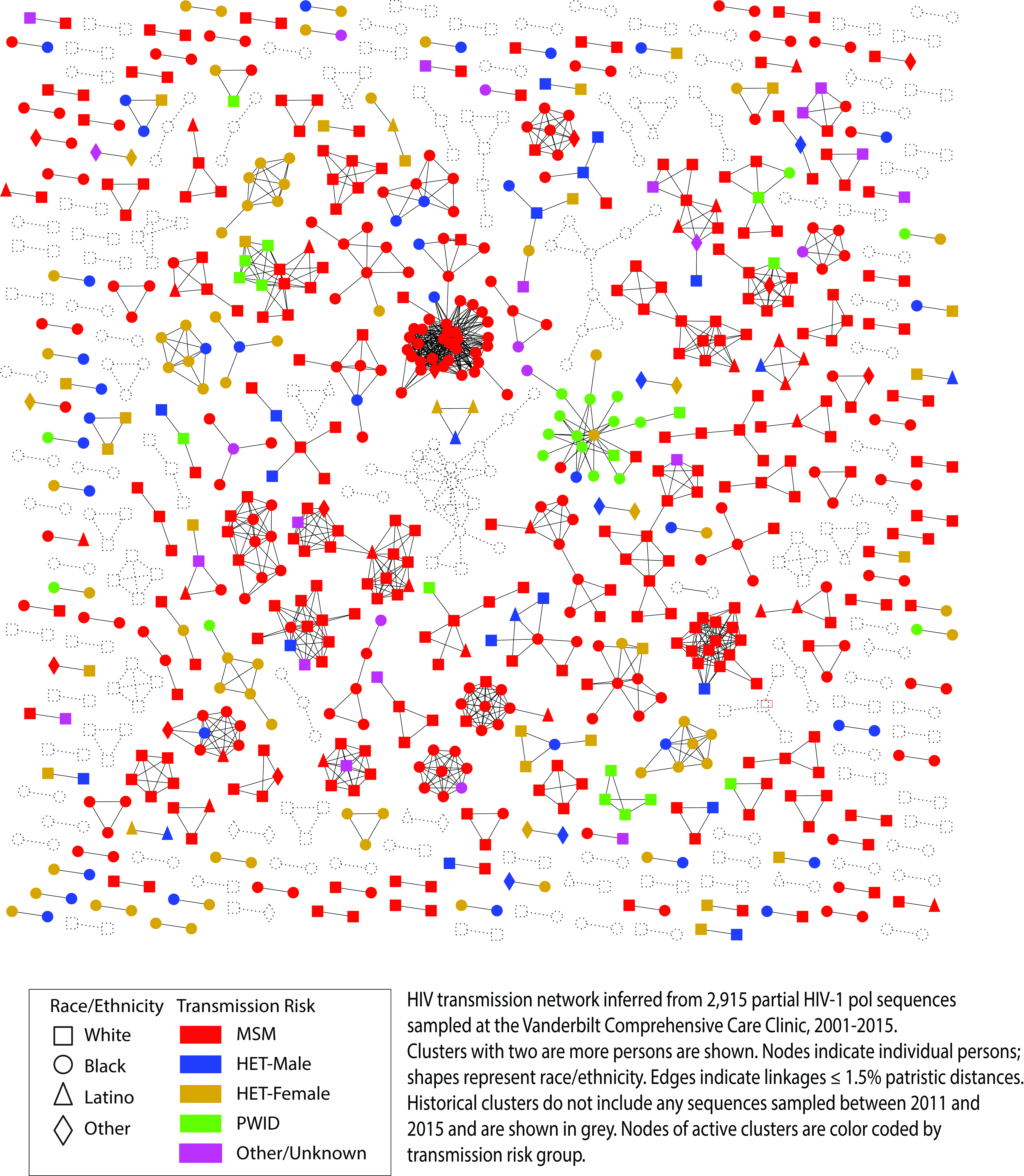

Supplement: Supplemental data [file Supp_Fig1.tif]
